# Association between combined urinary phthalate metabolites exposure and grip strength among residents in Guangzhou, China

**DOI:** 10.3389/fpubh.2025.1545872

**Published:** 2025-05-30

**Authors:** Jinbin Chen, Jie Shi, Guojun Xu, Wenru Feng, Jiayun Lv, Tongxing Shi, Qinqin Jiang

**Affiliations:** ^1^Guangzhou Key Laboratory for Clinical Rapid Diagnosis and Early Warning of Infectious Diseases, KingMed School of Laboratory Medicine, Guangzhou Medical University, Guangzhou, China; ^2^Guangzhou Center for Disease Control and Prevention, Guangzhou, China; ^3^The School of Public Health, Guangzhou Medical University, Guangzhou, China; ^4^Guangzhou Conghua District Center for Disease Control and Prevention, Guangzhou, China

**Keywords:** mPAEs, phthalates, grip strength, combined effects, BKMR

## Abstract

**Background:**

Exposure to environmental phthalate metabolites (mPAEs) has been suggested to potentially affect grip strength, either directly or indirectly. However, research on the impact of mPAEs mixtures on grip strength remains limited. This study aimed to investigate the independent and joint effects of co-exposure to multiple mPAEs on grip strength among residents of Guangzhou, China.

**Methods:**

Data were collected from 972 participants, and urinary concentrations of nine mPAEs (mMP, mEP, miBP, mnBP, mCHP, mEOHP, mEHHP, mBzP, and mEHP) were measured. To assess these relationships, we conducted generalized linear regression models, Bayesian Kernel Machine Regression (BKMR), and Weighted Quantile Sum (WQS) regression analyses.

**Results:**

The Results showed that higher quartiles of mMP, miBP, mCHP, mEHHP, and mEHP were associated with decreased grip strength compared to the first quartile (Q1): mMP (Q4 vs. Q1: *β* = −1.44, 95% CI: −2.65 to −0.23, *p* = 0.019); miBP (Q2 vs. Q1: *β* = −1.78, 95% CI: −2.956 to −0.61, *p* = 0.003; Q3 vs. Q1: *β* = −1.39, 95% CI: −2.57 to −0.21, *p* = 0.002; Q4 vs. Q1: *β* = −1.23, 95% CI: −2.43 to 0.03, *p* = 0.045); mCHP (Q2 vs. Q1: *β* = −1.20, 95% CI: −2.38 to −0.03, *p* = 0.043); mEHHP (Q3 vs. Q1: *β* = −1.34, 95% CI: −2.53 to −0.16, *p* = 0.026); and mEHP (Q4 vs. Q1: *β* = −1.20, 95% CI: −2.39 to −0.01, *p* = 0.049). Restricted cubic spline (RCS) analysis indicated that grip strength gradually decreased as exposure concentrations of mMP (P-overall = 0.004) and miBP (P-overall = 0.037) increased, while the relationship between mEHP (P-overall = 0.022, P-nonlinear = 0.022) and grip strength exhibited an inverted U-shape. BKMR model analysis revealed a significant negative correlation between co-exposure to urinary mPAEs and grip strength, with mMP being the most significant contributor.

**Conclusion:**

This study demonstrates that exposure to mPAEs mixtures is associated with decreased grip strength, particularly influenced by mMP. These findings underscore the necessity for further investigation into the underlying mechanisms and potential modifiers of this association.

## 1 Introduction

Grip strength serves as a critical indicator of upper limb muscle development and overall body strength, which tends to decline with age (1), and it has substantial predictive power for identifying high-risk populations (2). It is also associated with cognitive ability, mobility, functional status, and mortality rates (3–5). Lower grip strength correlates with higher incidence rates of cardiovascular disease, chronic obstructive pulmonary disease, and various cancers, including colorectal, lung, and breast cancer (6). Therefore, identifying risk factors that contribute to decreased grip strength is essential for preventing related diseases.

Phthalic acid esters (PAEs), commonly known as phthalates, are a class of chemicals derived from phthalic acid that are readily metabolized into phthalate metabolites (mPAEs) in the human body (7). Classified by molecular weight into high and low categories, PAEs have distinct applications as plasticizers and softeners in numerous consumer products such as personal care items, children’s toys, food packaging, pharmaceuticals, textiles, medical devices, decorative materials, and building supplies (8, 9). Due to non-covalent bonds with their matrices, PAEs can easily migrate and volatilize from products, leading to widespread environmental exposure and contamination (10). These compounds are predominantly released into the environment through industrial production and consumer product effluents, and their metabolites have been detected in various human bodily fluids, drawing significant attention to their health risks (11, 12).

A study in South Korea involving 1,228 participants showed negative correlations between concentrations of mono-(2-ethyl-5-oxohexyl) phthalate (mEOHP), mono-(2-ethyl-5-hydroxyhexyl) phthalate (mEHHP), and mono-n-butyl phthalate (mnBP) and grip strength (13). A U.S. cross-sectional analysis indicated that phthalate exposure was negatively associated with grip strength among adults, irrespective of gender or age (14). However, these studies primarily focused on individual mPAE effects. In reality, populations are often exposed to multiple mPAEs simultaneously, which may exert antagonistic, synergistic, or additive effects on health. Thus, evaluating the overall impact of mixed mPAEs on grip strength and potential interactions among mPAEs is crucial. Furthermore, to the best of our knowledge, no previous studies have examined the effects of mPAEs exposure on grip strength specifically within the Chinese population.

This study aims to assess the association between co-exposure to multiple mPAEs and grip strength using data from residents in Guangzhou, China. Employing generalized linear regression, Bayesian Kernel Machine Regression (BKMR), and Weighted Quantile Sum (WQS) regression analyses, this research tests the hypothesis that exposure to a mixture of mPAEs may be associated with decreased grip strength.

## 2 Materials and methods

### 2.1 Study population

This study is part of the broader Guangzhou human biomonitoring program. The biomonitoring study aimed to encompass all randomly selected participants across a wide age spectrum. This study was conducted in three districts of Guangzhou, China: Yuexiu District, Panyu District, and Conghua District. Six community-based survey units were randomly selected from each district. Within each survey unit, participants were stratified by age and gender, and individuals were randomly selected as subjects. Inclusion criteria: residents who had lived in the selected survey unit for at least 6 months prior to the survey. Exclusion criteria: (1) individuals with cognitive impairments. (2) Individuals with a history of severe illnesses such as cancer. (3) Participants unable to perform grip strength testing (e.g., infants, or individuals with physical impairments). After excluding participants with missing data, a total of 972 individuals were included in the final analysis, ranging in age from 14 to 79 years. A pre-designed questionnaire was administered to collect health and environmental exposure information.

### 2.2 Measurement of urinary mPAEs and grip strength

Urine samples were collected to analyze the concentrations of nine phthalate metabolites (mPAEs), including monomethyl phthalate (mMP), monoethyl phthalate (mEP), mono-isobutyl phthalate (miBP), mono-n-butyl phthalate (mnBP), monocyclohexyl phthalate (mCHP), mono-(2-ethyl-5-oxohexyl) phthalate (mEOHP), mono-(2-ethyl-5-hydroxyhexyl) phthalate (mEHHP), monobenzyl phthalate (mBzP), and mono (2-ethylhexyl) phthalate (mEHP). The analysis of these metabolites was performed using ultra-high performance liquid chromatography–tandem mass spectrometry (UHPLC–MS/MS). During the health examination, a midstream urine sample of at least 40 mL was collected from each participant. All urine samples were delivered to the laboratory for analysis within 4 h of collection. Grip strength was measured using a standard-sized hydraulic hand dynamometer, with participants instructed to perform the test according to standardized protocols to ensure consistency and accuracy. Grip strength was measured using an electronic grip strength tester (AP-1005, AOPI, China).

### 2.3 Covariates

In this study, a comprehensive set of covariates was included to control for potential confounding factors based on previous literature (3, 6, 13–15), encompassing demographic, socioeconomic, lifestyle, and health-related variables: age, sex (male, female), race/ethnicity (Han ethnicity, other), marital status (never married, married, divorced/widowed), educational level (junior high school or lower, senior high school or technical secondary school, college or higher), income (<100,000 RMB, ≥100,000 RMB), body mass index (BMI) (<24.0 kg/m^2^, 24.0–27.9 kg/m^2^, ≥28.0 kg/m^2^), drinking history (never, current, ever), smoking history (never, current, ever), physical activity (never exercise, exercise 3 days per month, exercise 1–2 days per week, exercise 3–4 days per week, exercise 5–7 days per week), fracture condition (no, yes), specifically current or recent fractures (i.e., within the past 6 months) in the upper limbs, and chronic diseases (no, yes). The chronic diseases considered in this study include hypertension, diabetes, coronary heart disease, stroke, chronic obstructive pulmonary disease (COPD), asthma, chronic hepatitis or liver cirrhosis, and peptic ulcer.

### 2.4 Statistical analysis

In the descriptive analysis, continuous variables were summarized using mean (±SD), while categorical variables were described using frequency counts and percentages n (%). Given that the concentrations of mPAEs in urine did not follow a normal distribution as indicated by normality tests, we adjusted the mPAEs concentrations for urinary creatinine and subsequently performed logarithmic transformation. The transformed mPAEs concentrations were then categorized into quartiles based on the 25th, 50th, and 75th percentiles (Q1: reference, Q2, Q3, and Q4). To explore the relationships between different mPAEs in urine, Spearman correlation analysis was conducted.

Generalized linear regression models were employed to evaluate the association between each type of urinary mPAE and grip strength, incorporating median concentrations for trend testing. Results were presented as *β* coefficients with corresponding 95% confidence intervals (CI). All models were adjusted for covariates including age, sex, education level, income, race/ethnicity, marital status, BMI, drinking history, smoking history, physical activity, fracture condition, and chronic diseases. Additionally, restricted cubic spline (RCS) analysis was used to assess potential nonlinear relationships between each mPAE and grip strength. In the RCS analysis, the median concentration served as the reference point, with knots set at the 10th, 50th, and 90th percentiles of the mPAE distribution.

To investigate the overall impact of mPAEs mixtures on grip strength, Bayesian Kernel Machine Regression (BKMR) models were applied (16). This approach employs a Gaussian kernel function for the iterative regression of the exposure-response relationship, obviating the need to define a specific parametric form. Model construction was achieved through posterior sampling using the Markov Chain Monte Carlo (MCMC) algorithm. The model equation is structured as follows:

$$Y_{i}=h\;(Z_{i1}+Z_{i2}+\cdots +Z_{\mathrm{iM}})+x_{i}^{\prime }\;\beta +\in_{i}$$

$Y_{i}$ denotes the grip strength of the $i^{\mathrm{th}}$ individual ( i = 1,… n); ℎ() represents the function (kernel function) utilized for fitting the effect of mPAEs exposure on grip strength; *M* indicates the $M^{\mathrm{th}}$ type of mPAEs exposure; $x_{i}^{\prime }$ and 𝛽 denote the potential covariates and their corresponding effects, respectively; $\in_{i}$ represents residuals that follow the $\in_{j}$ ~𝑁 (0, 𝜎^2^) distribution. We evaluated the estimated effects of a 5% increase or decrease in the median concentration of individual mPAEs. The non-linear dose–response relationship for each mPAE was also assessed. BKMR models were run for 20,000 iterations, estimating posterior inclusion probabilities (PIPs) for each mPAE to determine its relative importance in influencing grip strength.

Furthermore, Weighted Quantile Sum (WQS) regression models were utilized to evaluate the combined effect of exposure to mPAEs mixtures on grip strength. This method integrates weighted quantile sum approaches with linear regression to construct a weighted index that examines the association between this index and the outcome variable (17, 18). The model equation is structured as follows:

$$\begin{array}{l} g(\mu )=\beta_{0}+\beta_{1}\;\sum_{i=1}^{a}w_{i}q_{i}+z\prime \varphi \;\\ \mathrm{WQS}=\sum_{i=1}^{a}w_{i}q_{i} \end{array}$$

The function 𝑔 (𝜇) is specified as a linear link function (family = Gaussian); $\beta_{0}$ represents the intercept; z′ denotes the matrix of covariates; 𝜑 indicates the coefficients for the covariates; $\beta_{1}$ represents the regression coefficient for WQS; a signifies the total number of mPAEs exposures considered; $w_{i}$ denotes the weight for each type of mPAE, with the sum of all mPAEs’ weights being equal to one; $q_{i}$ represents the quantile for each type of mPAE, which in this study is set to $q_{i}$ = 4. By assigning a weighted index (ranging from 0 to 1) to each mPAE exposure variable, we assessed the relative importance of individual mPAEs on grip strength. The WQS weighted index was derived through 10,000 iterations using the R package “gWQS.”

All data analyses were conducted using R software (version 4.3.2). Statistical significance was set at *p* < 0.05.

## 3 Results

### 3.1 Study population characteristics

The general characteristics of the study population, stratified by quartiles of grip strength, are summarized in Table 1. This study included a total of 972 participants, with 392 (40.3%) being male and 580 (59.7%) female. Significant differences were observed among the four grip strength quartile groups regarding age, sex, educational level, marital status, alcohol consumption history, smoking status, and presence of chronic diseases (all *p* < 0.05). Specifically, individuals who were younger, male, had higher levels of education, were married, currently consumed alcohol, currently smoked, and did not report chronic diseases tended to exhibit greater grip strength. Conversely, older participants, females, those with lower educational attainment, unmarried individuals, non-drinkers, non-smokers, and those with chronic diseases were more likely to be in the lower grip strength quartiles.

**Table 1:** Characteristics of the study population.

Characteristics of the study population	Total	Grip				*p*-value
		Q1	Q2	Q3	Q4	
	(*N* = 972)	(*N* = 247)	(*N* = 240)	(*N* = 242)	(*N* = 243)	
Age						
Mean (SD)	52.0 (17.0)	57.5 (17.5)	51.5 (16.3)	50.8 (17.1)	48.0 (15.5)	<0.001
Gender						
Male	392 (40.33%)	26 (10.53%)	28 (11.67%)	107 (44.21%)	231 (95.06%)	<0.001
Female	580 (59.67%)	221 (89.47%)	212 (88.33%)	135 (55.79%)	12 (4.94%)	
Education level						
Junior high school or lower	426 (43.83%)	145 (58.70%)	104 (43.33%)	102 (42.15%)	75 (30.86%)	<0.001
Senior high school or technical secondary school	293 (30.14%)	66 (26.72%)	74 (30.83%)	72 (29.75%)	81 (33.33%)	
College or higher	253 (26.03%)	36 (14.57%)	62 (25.83%)	68 (28.10%)	87 (35.80%)	
Income						
<100,000 RMB/year	326 (33.54%)	90 (36.44%)	76 (31.67%)	81 (33.47%)	79 (32.51%)	0.840
≥100,000 RMB/year	646 (66.46%)	157 (63.56%)	164 (68.33%)	161 (66.53%)	164 (67.49%)	
Race/ethnicity						
Han ethnicity	965 (99.28%)	247 (100.00%)	238 (99.17%)	241 (99.59%)	239 (98.35%)	0.280
Other	7 (0.72%)	0 (0.00%)	2 (0.83%)	1 (0.41%)	4 (1.65%)	
Marital status						
Never married	122 (12.55%)	24 (9.72%)	27 (11.25%)	36 (14.88%)	35 (14.40%)	<0.001
Married	784 (80.66%)	183 (74.09%)	198 (82.50%)	201 (83.06%)	202 (83.13%)	
Divorced/Widowed	66 (6.79%)	40 (16.19%)	15 (6.25%)	5 (2.07%)	6 (2.47%)	
BMI						
<24.0	566 (58.23%)	153 (61.94%)	146 (60.83%)	140 (57.85%)	127 (52.26%)	0.469
24.0 ~ 27.9	290 (29.84%)	63 (25.51%)	72 (30.00%)	73 (30.17%)	82 (33.74%)	
≥28.0	116 (11.93%)	31 (12.55%)	22 (9.17%)	29 (11.98%)	34 (13.99%)	
Drinking history						
Never	826 (84.98%)	240 (97.17%)	223 (92.92%)	204 (84.30%)	159 (65.43%)	<0.001
Current	126 (12.96%)	5 (2.02%)	14 (5.83%)	32 (13.22%)	75 (30.86%)	
Ever	20 (2.06%)	2 (0.81%)	3 (1.25%)	6 (2.48%)	9 (3.70%)	
Smoking history						
Never	778 (80.04%)	236 (95.55%)	223 (92.92%)	187 (77.27%)	132 (54.32%)	<0.001
Current	157 (16.15%)	7 (2.83%)	12 (5.00%)	41 (16.94%)	97 (39.92%)	
Ever	37 (3.81%)	4 (1.62%)	5 (2.08%)	14 (5.79%)	14 (5.76%)	
Physical activity						
Never exercise	73 (7.51%)	17 (6.88%)	15 (6.25%)	27 (11.16%)	14 (5.76%)	0.135
Exercise 3 days per month	43 (4.42%)	8 (3.24%)	5 (2.08%)	15 (6.20%)	15 (6.17%)	
Exercise 1–2 days per week	204 (20.99%)	44 (17.81%)	53 (22.08%)	44 (18.18%)	63 (25.93%)	
Exercise 3–4 days per week	123 (12.65%)	32 (12.96%)	28 (11.67%)	35 (14.46%)	28 (11.52%)	
Exercise 5–7 days per week	529 (54.42%)	146 (59.11%)	139 (57.92%)	121 (50.00%)	123 (50.62%)	
Fracture condition						
No	962 (98.97%)	245 (99.19%)	237 (98.75%)	242 (100.00%)	238 (97.94%)	0.260
Yes	10 (1.03%)	2 (0.81%)	3 (1.25%)	0 (0.00%)	5 (2.06%)	
Chronic disease						
No	706 (72.63%)	168 (68.02%)	168 (70.00%)	173 (71.49%)	197 (81.07%)	0.015
Yes	266 (27.37%)	79 (31.98%)	72 (30.00%)	69 (28.51%)	46 (18.93%)	

BMI, body mass index (calculated as weight in kilograms divided by height in meters squared).

### 3.2 Urine mPAEs distribution and correlations

**Figure 1:**
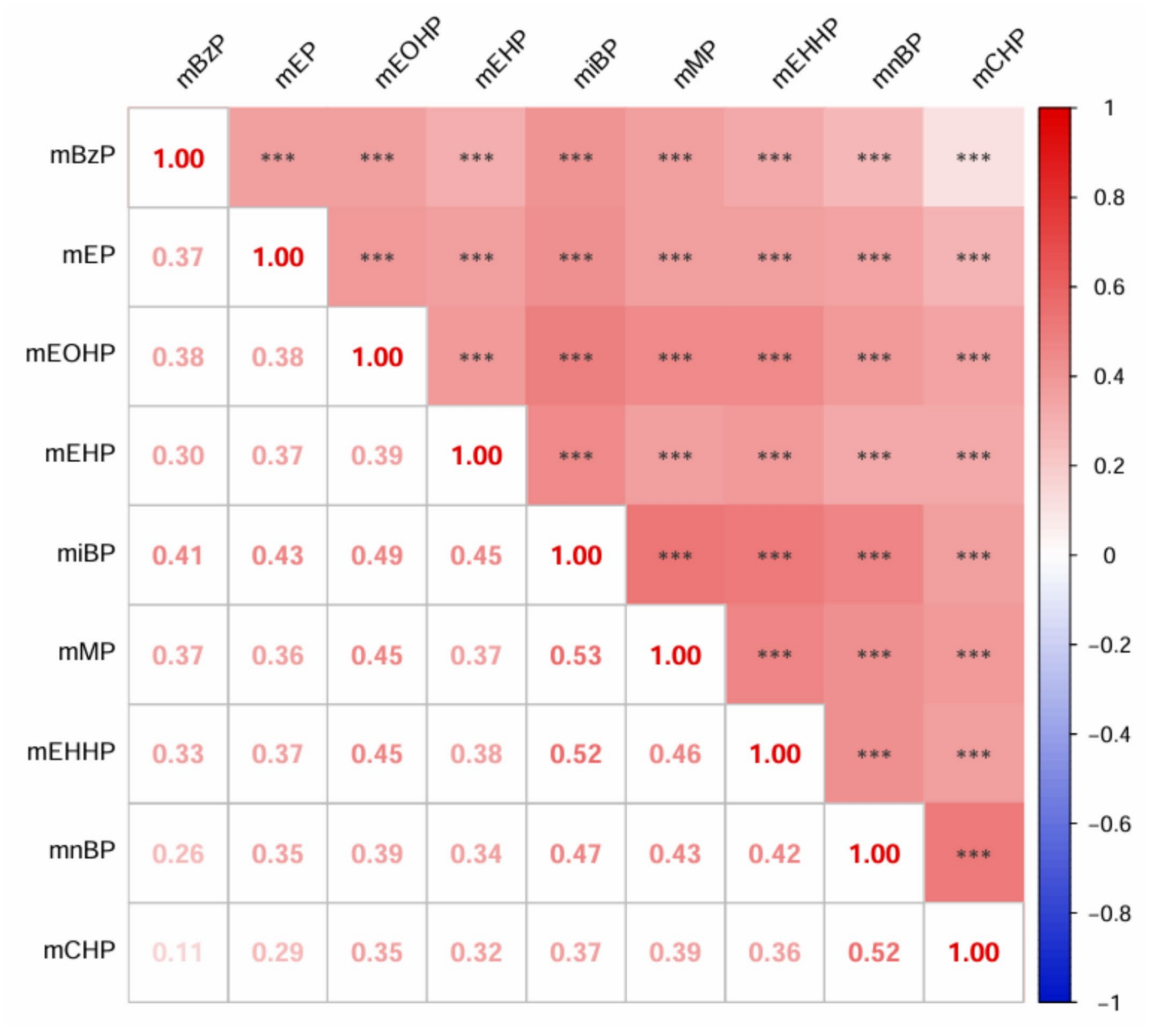
Spearman’s correlation matrix among ln-transformed urinary mPAEs in the study population. All the correlations were statistically significant (***p < 0.001).

As detailed in Supplementary Table S1, the distribution of mPAEs concentrations is presented, with values expressed as μg/g creatinine. Among the measured compounds, mnBP exhibited the highest concentration, with a mean of 150.78 ± 204.12 μg/g creatinine, followed by miBP, which had a mean concentration of 82.41 ± 95.15 μg/g creatinine. Conversely, mCHP showed the lowest concentration, with a mean of 0.43 ± 0.84 μg/g creatinine. The results of the Spearman correlation analysis for mPAEs are depicted in Figure 1. These findings indicate significant positive correlations between urinary mPAEs concentrations. Specifically, mMP demonstrated moderate correlations with both mEHHP (r = 0.53, *P <* 0.001) and miBP (r = 0.52). Additionally, mCHP was moderately correlated with mnBP (r = 0.52).

### 3.3 Generalized linear regression analysis of mPAEs and grip strength

**Figure 2:**
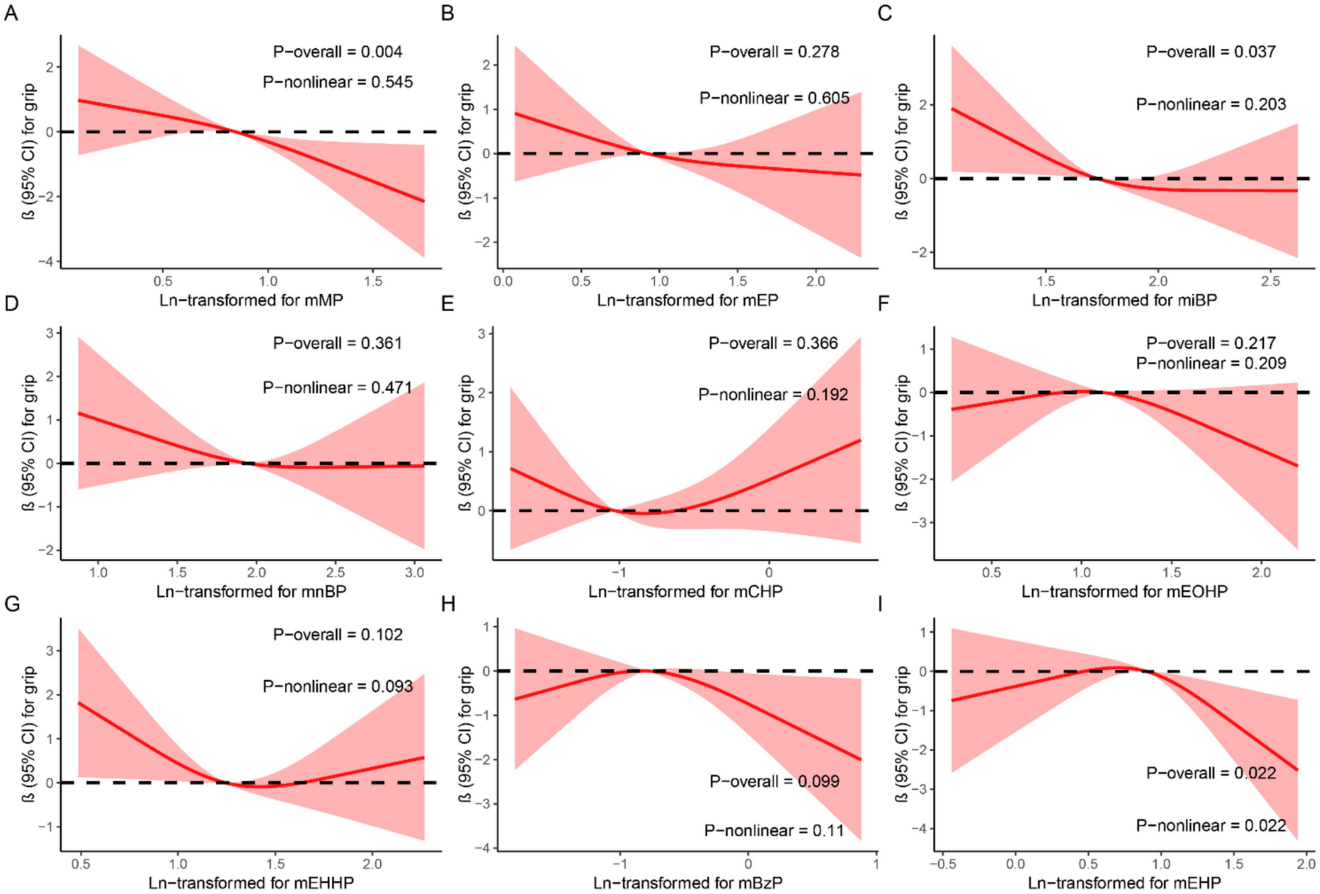
Dose–response relationship between urinary mPAEs and grip strength were estimated by RCS models. Models were adjusted for age, sex, education level, income, race/ethnicity, marital status, BMI, drinking history, smoking history, physical activity, fracture condition, and chronic disease. **(A)** mMP; **(B)** mEP; **(C)** miBP; **(D)** mnBP; **(E)** mCHP; **(F)** mEOHP; **(G)** mEHHP; **(H)** mBzP; **(I)** mEHP.

In the generalized linear regression analysis, each mPAEs concentration was log-transformed to fit a normal distribution. After adjusting for all covariates, we found that compared to the first quartile (Q1), higher quartiles of mMP, miBP, mCHP, mEHHP, and mEHP were associated with decreased grip strength: mMP (Q4 vs. Q1: *β* = −1.44, 95% CI: −2.65 to −0.23, *p* = 0.019); miBP (Q2 vs. Q1: *β* = −1.78, 95% CI: −2.956 to −0.61, *p* = 0.003; Q3 vs. Q1: *β* = −1.39, 95% CI: −2.57 to −0.21, *p* = 0.002; Q4 vs. Q1: *β* = −1.23, 95% CI: −2.43 to 0.03, *p* = 0.045); mCHP (Q2 vs. Q1: *β* = −1.20, 95% CI: −2.38 to −0.03, *p* = 0.043); mEHHP (Q3 vs. Q1: *β* = −1.34, 95% CI: −2.53 to −0.16, *p* = 0.026); and mEHP (Q4 vs. Q1: *β* = −1.20, 95% CI: −2.39 to −0.01, *p* = 0.049). Conversely, mBzP showed an increase in grip strength (Q2 vs. Q1: *β* = 1.50, 95% CI: 0.35 to 2.66, *p* = 0.011) (Table 2). Figure 2 illustrates the results of RCS analyses, revealing that increased exposure concentrations of mMP (P-overall = 0.004) and miBP (P-overall = 0.037) were associated with a decline in grip strength. Additionally, a nonlinear relationship between mEHP and grip strength was observed, following an inverted U-shape where grip strength initially increases with rising mEHP exposure before subsequently decreasing (P-overall = 0.022, P-nonlinear = 0.022).

**Table 2:** The association between mPAEs and grip strength.

Urinary phthalate metabolites	*β* coefficient	95% CI	*P-*value
mMP			
Q1	Reference	Reference	–
Q2	−0.569	(−1.741, 0.602)	0.341
Q3	−0.758	(−1.935, 0.419)	0.207
Q4	−1.444	(−2.651, −0.236)	0.019
mEP			
Q1	Reference	Reference	–
Q2	−0.193	(−1.352, 0.966)	0.744
Q3	−0.622	(−1.796, 0.552)	0.300
Q4	−0.642	(−1.821, 0.537)	0.286
miBP			
Q1	Reference	Reference	–
Q2	−1.786	(−2.956, −0.617)	0.003
Q3	−1.391	(−2.572, −0.211)	0.021
Q4	−1.233	(−2.437, −0.030)	0.045
mnBP			
Q1	Reference	Reference	–
Q2	−0.079	(−1.252, 1.094)	0.895
Q3	−0.157	(−1.324, 1.010)	0.792
Q4	−0.635	(−1.823, 0.553)	0.295
mCHP			
Q1	Reference	Reference	–
Q2	−1.209	(−2.379, −0.039)	0.043
Q3	−0.927	(−2.111, 0.257)	0.125
Q4	−0.006	(−1.209, 1.196)	0.992
mEOHP			
Q1	Reference	Reference	–
Q2	0.310	(−0.862, 1.482)	0.604
Q3	0.413	(−0.772, 1.599)	0.495
Q4	−0.696	(−1.897, 0.504)	0.256
mEHHP			
Q1	Reference	Reference	–
Q2	−1.133	(−2.305, 0.038)	0.058
Q3	−1.348	(−2.530, −0.166)	0.026
Q4	−0.889	(−2.085, 0.308)	0.146
mBzP			
Q1	Reference	Reference	–
Q2	1.509	(0.352, 2.666)	0.011
Q3	0.170	(−1.000, 1.340)	0.776
Q4	−0.202	(−1.382, 0.977)	0.737
mEHP			
Q1	Reference	Reference	–
Q2	−0.101	(−1.263, 1.062)	0.865
Q3	−0.206	(−1.377, 0.965)	0.730
Q4	−1.203	(−2.398, −0.008)	0.049

The models were adjusted by age, sex, race/ethnicity, marital status, educational level, income, BMI, drinking history, smoking history, physical activity, fracture condition and chronic diseases.

### 3.4 BKMR analysis of mPAEs mixtures and grip strength

**Figure 3:**
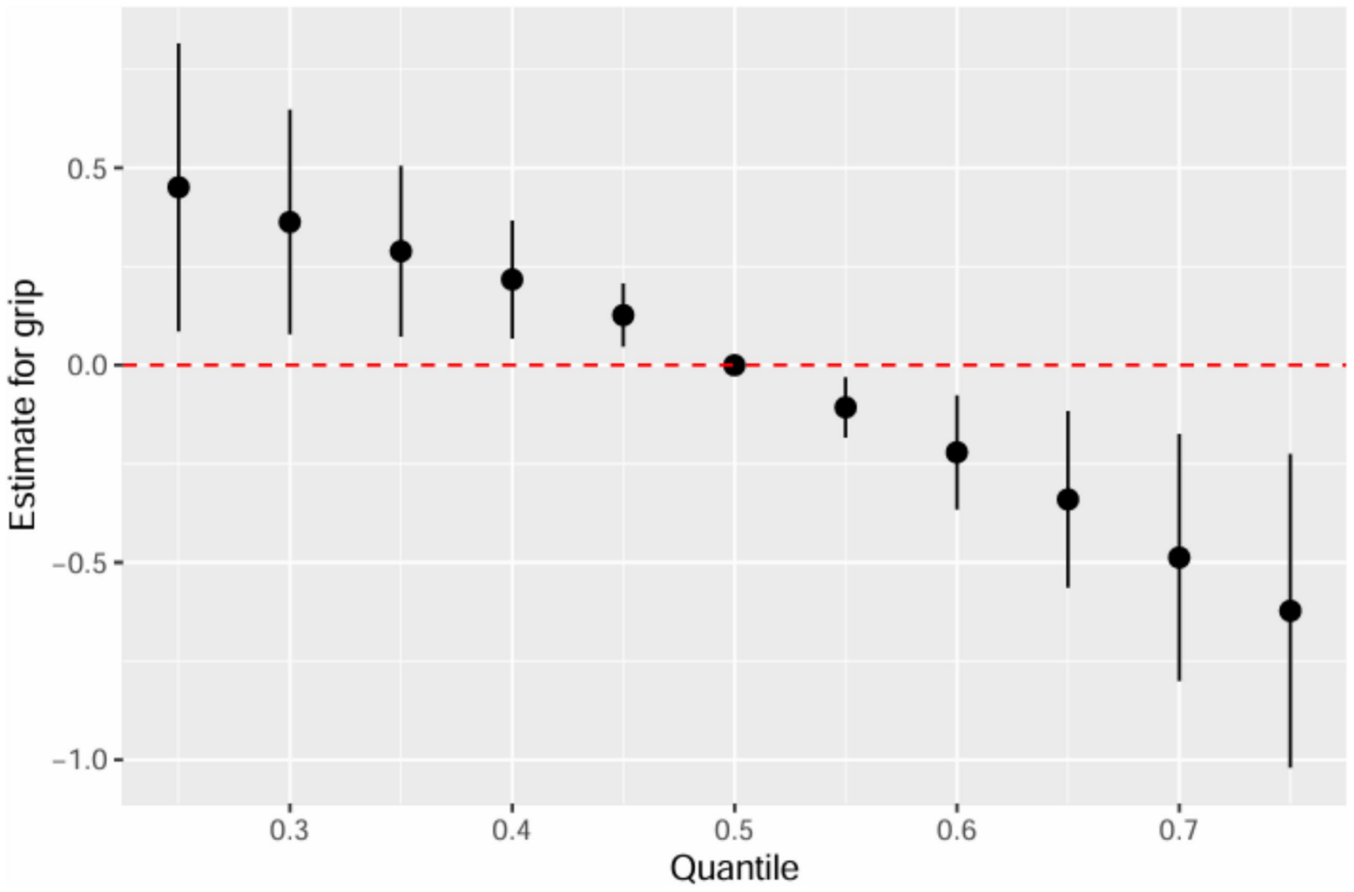
Joint effect (95% CI) of the mPAEs mixture on grip strength by using BKMR models, when all ln-transformed mPAEs concentrations at particular percentiles were compared to all ln-transformed mPAEs concentrations at their 50th percentile. The models were adjusted for age, sex, education level, income, race/ethnicity, marital status, BMI, drinking history, smoking history, physical activity, fracture condition, and chronic disease.

**Figure 4:**
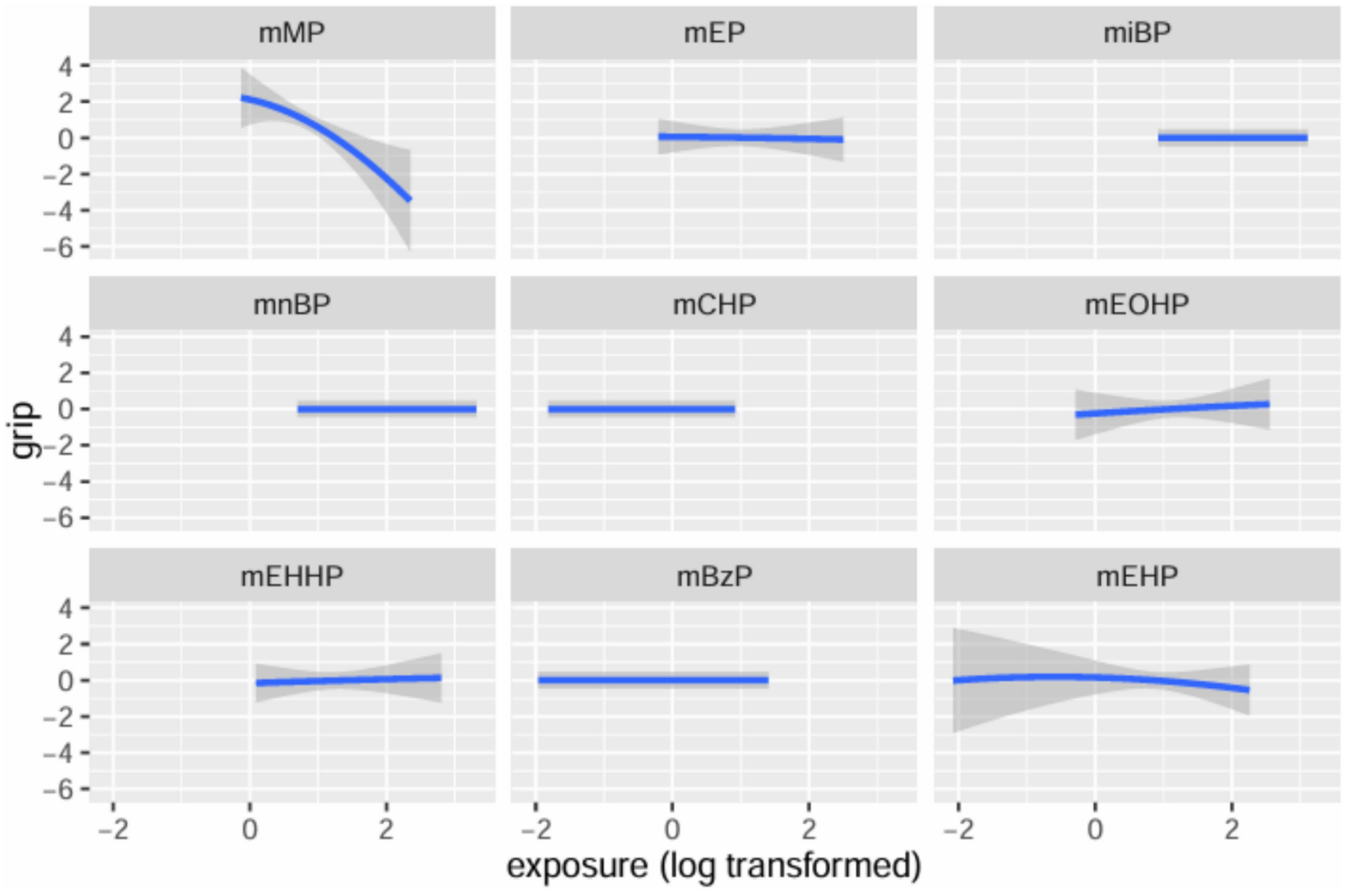
Univariate exposure-response functions and 95% confidence interval for each mPAEs with the other mPAEs fixed at the median.

Table 3 summarizes the PIPs for each mPAE in the BKMR model. Among these, mMP demonstrated the highest contribution to the model with a PIP of 0.980, followed by mEHP, which had a PIP of 0.112. The overall impact of the mPAEs mixture on grip strength is illustrated in Figure 3. The analysis revealed a significant negative association between higher urinary levels of the mPAEs mixture and decreased grip strength when concentrations reached or exceeded the 55th percentile compared to the 50th percentile. To investigate potential nonlinear relationships, exposure-response functions were estimated for each mPAE while holding all other mPAEs at their median values. These results are presented in Figure 4. Specifically, increasing exposure to mMP was associated with a progressive reduction in grip strength. In contrast, there was a slight negative trend in grip strength with increased exposure to mEHP, whereas a slight positive trend was noted with increasing exposure to mEOHP.

**Table 3:** Posterior inclusion probabilities (PIPs) of mPAEs in the BKMR model.

mPAEs	Grip
mMP	0.980
mEP	0.020
miBP	0.000
mnBP	0.000
mCHP	0.000
mEOHP	0.026
mEHHP	0.022
mBzP	0.000
mEHP	0.112

### 3.5 WQS analysis of mPAEs mixtures and grip strength

**Figure 5:**
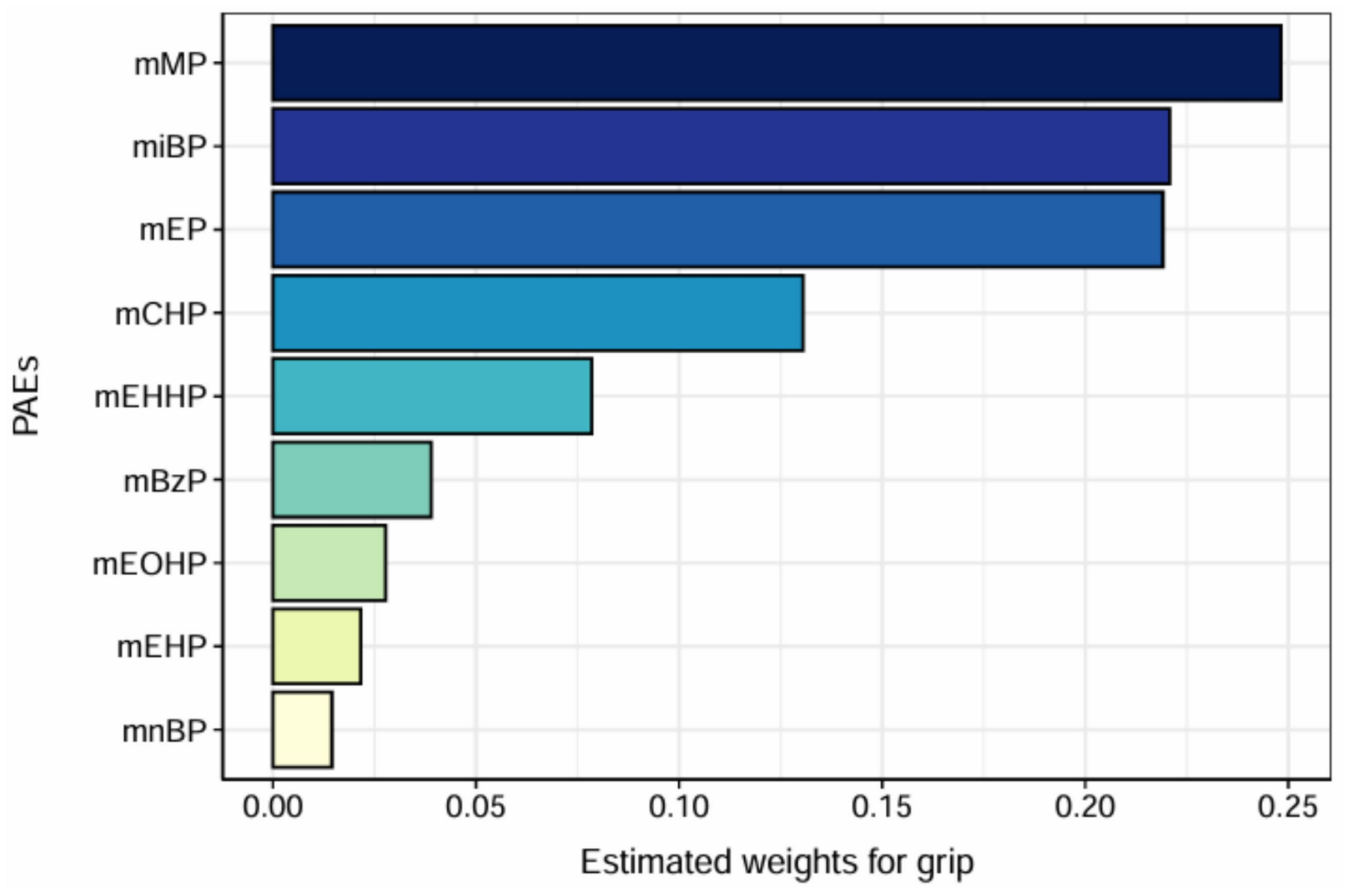
WQS model regression index weights. Models were adjusted for age, sex, education level, income, race/ethnicity, marital status, BMI, drinking history, smoking history, physical activity, fracture condition, and chronic disease.

We utilized WQS regression analysis to evaluate the potential negative association between mixed exposure to mPAEs and grip strength. The estimated weights of individual mPAEs contributing to the WQS index are presented in Figure 5. Among these, monomethyl phthalate (mMP) exhibited the highest weight at 0.248, followed by mono-isobutyl phthalate (miBP) with a weight of 0.221, and monoethyl phthalate (mEP) with a weight of 0.219.Although the overall WQS index did not show a statistically significant relationship with grip strength (t = −1.120, *p* = 0.262), the results suggest that mMP and miBP were the most heavily weighted contributors within the mixed exposure. This indicates that these two metabolites may play a more prominent role in the potential impact on grip strength.

## 4 Discussion

This study employed a comprehensive statistical framework, incorporating generalized linear regression models, BKMR, and WQS models, to evaluate both the independent and joint effects of urinary mPAEs mixtures on grip strength. Our findings indicate significant associations between co-exposure to urinary mPAEs and grip strength. Specifically, generalized linear regression models revealed that mMP, miBP, mCHP, and mEHP were significantly negatively associated with grip strength. Restricted cubic spline (RCS) analysis further demonstrated dose–response relationships for mMP and mEHP. In the BKMR model, we observed a significant negative correlation between exposure to urinary mPAEs mixtures and grip strength, with mMP identified as the primary contributing factor. The WQS model suggested that mMP may be the most influential component within the mixture, despite the lack of overall statistical significance. This suggests that mMP may play a prominent role in the potential impact of mPAEs on grip strength.

Numerous studies have consistently demonstrated significant associations between phthalate exposure and adverse health outcomes, including cardiovascular disease, reproductive disorders, various types of cancer, and metabolic syndromes such as obesity and diabetes (19–22). Grip strength has also been recognized as a significant biomarker for various adverse health outcomes, demonstrating predictive validity for short-term and long-term mortality and morbidity. Diminished grip strength in ostensibly healthy adults is associated with an elevated risk of functional constraints, disability in later life stages, and increased all-cause mortality (23). Previous research has shown that mEHP exposure is negatively associated with grip strength in Korea population, consistent with our study (13).

Our study explores the negative impacts of both individual mPAEs and their mixtures on grip strength within the Chinese Guangzhou population, a relationship that has not been previously investigated. These observations suggest that considering multiple phthalate metabolites simultaneously may be valuable when assessing their impact on muscle function. However, the study population recruited from Guangzhou, may not be fully representative of the broader Chinese population. Guangzhou, a highly urbanized and industrialized city in southern China, likely has unique environmental and socioeconomic characteristics that influence mPAEs exposure levels. Previous studies have observed regional variations in phthalate exposure across China, with higher urinary concentrations of certain mPAEs, such as monoethyl phthalate (MEP) and monobutyl phthalate (MBP), reported in urban areas like Guangzhou compared to rural regions (24). These differences may be attributed to variations in industrial activity, consumer product use, and dietary patterns. For example, urban populations may have greater exposure to phthalate-containing products, such as plastics and personal care products, compared to rural populations (25). Additionally, in this study cohort, 99.28% of the participants are of Han Chinese ethnicity, which could restrict the generalizability of our findings to other ethnic groups. This is because the relationship between phthalate exposure and grip strength may be influenced by genetic, environmental, and lifestyle factors that vary across populations. Furthermore, regional differences in lifestyle factors, such as physical activity or occupational exposures, could modulate the relationship between mPAEs exposure and grip strength. Therefore, our findings may not be generalizable to all regions of China, particularly less industrialized areas. Future studies should include diverse populations from multiple regions to better characterize the association between mPAEs exposure and health outcomes across China.

U-shaped dose–response relationships have been consistently and independently observed across a wide range of biological, toxicological, and pharmacological studies (26). In this studies, the RCS analyses revealed distinct dose–response relationships for mPAEs, with mMP and miBP showing generally linear negative associations with grip strength, while mEHP exhibited an inverted U-shaped pattern, indicating a non-linear relationship. Besides, we also observed that compared to the low concentration (Q1), an unexpected positive association was observed for mBzP in the second quartile (Q2). This unexpected trend for mEHP and mBzP, where grip strength initially increases at low to moderate exposure levels before declining at higher levels, may reflect complex biological mechanisms. One plausible explanation is a hormetic response, where low-dose exposure to mEHP might trigger adaptive mechanisms, such as upregulation of antioxidant defenses, that temporarily enhance muscle function, while higher doses overwhelm these defenses, leading to oxidative stress and muscle damage (38). Alternatively, mEHP, a metabolite of di-(2-ethylhexyl) phthalate (DEHP), may interact with receptors like PPARα at varying concentrations, with saturation at higher exposure levels altering metabolic pathways in muscle tissue. Differential metabolism or detoxification of mEHP at higher exposure levels could also contribute to this non-linear pattern (27). However, the wide confidence intervals at higher mPAEs concentrations suggest uncertainty in this trend, potentially due to limited sample size or exposure variability. The inverted U-shaped association warrants cautious interpretation. Future studies should employ larger cohorts and mechanistic experiments, to validate this non-linear relationship and elucidate the underlying pathways, including oxidative stress, inflammation, or receptor-mediated effects.

Both the BKMR and WQS models identified mMP as the strongest contributor to decreased grip strength, consistent with its high prevalence and potency in our study population. However, the effects of mPAEs on grip strength differ between the two models. For instance, the WQS analysis does not indicate an overall statistically significant association, which is likely attributable to methodological differences. The BKMR model captures non-linear relationships and potential interactions among mPAEs, allowing for a more flexible estimation of their combined and individual effects on grip strength (16). For instance, BKMR may detect synergistic or antagonistic interactions that influence the apparent effect sizes of certain mPAEs. In contrast, the WQS model assumes linear and additive effects, assigning weights to each mPAE based on its contribution to the outcome while constraining effects to a single direction (18). This assumption may oversimplify the complex exposure mixture, potentially leading to differences in the estimated effects of mPAEs. These findings underscore the importance of using complementary statistical approaches to analyze environmental mixtures and suggest that future studies should further explore non-linear and interactive effects of mPAEs to refine our understanding of their impact on health outcomes.

Phthalates, a class of chemicals widely used as plasticizers in plastic products, have been increasingly associated with various adverse health outcomes, including reduced grip strength, an important indicator of muscle strength and function (14). The decline in grip strength likely reflects impaired muscle quality and functionality, mediated through multiple biological mechanisms that directly or indirectly compromise muscle performance. Phthalates are recognized as endocrine-disrupting chemicals capable of mimicking or interfering with endogenous hormones, particularly sex hormones and thyroid hormones. Notably, phthalate exposure has been linked to decreased testosterone levels, a critical hormone for maintaining muscle mass and strength. Meeker et al. (28) reported a negative correlation between urinary phthalate metabolite concentrations and serum testosterone levels, suggesting that reduced testosterone may impair muscle protein synthesis, thereby diminishing muscle strength and grip strength (28). Zhao et al. (29) found that ferroptosis is a key factor in the decrease of testosterone levels induced by phthalates. Additionally, phthalate exposure may compromise muscle function by inducing oxidative stress and inflammation. Oxidative stress, characterized by excessive production of reactive oxygen species (ROS), can damage cellular components, including mitochondrial and nuclear DNA in muscle cells, leading to reduced muscle strength (30, 31). Ferguson et al. (32) demonstrated that phthalate exposure is associated with elevated oxidative stress biomarkers. It may impair mitochondrial function in muscle cells, thereby limiting energy production and contributing to muscle weakness. Furthermore, heightened inflammatory responses, as evidenced by elevated C-reactive protein (CRP) levels, are negatively associated with grip strength, indicating that inflammation may be a key contributor to muscle dysfunction (33, 34). Phthalates may exacerbate inflammation by suppressing peroxisome proliferator-activated receptor-*γ* (PPAR-γ) activity, which normally mitigates inflammation by reducing pro-inflammatory cytokine production (27). This inflammatory milieu may disrupt muscle protein metabolism, further compromising muscle strength. Phthalate exposure is also associated with metabolic disturbances, such as insulin resistance and lipid metabolism dysregulation, which may indirectly impair muscle function. Insulin resistance can reduce glucose uptake in muscle cells, limiting energy availability for muscle contraction. Stahlhut et al. (35) found that phthalate exposure is linked to increased waist circumference and insulin resistance, which may exacerbate muscle dysfunction (36). In summary, the association between phthalate exposure and reduced grip strength likely involves a complex interplay of mechanisms, including endocrine disruption, oxidative stress, inflammation, and metabolic dysregulation. These pathways may interact synergistically to impair muscle function. Future research should elucidate the precise contributions of these mechanisms and explore interventions to mitigate the adverse effects of phthalates on muscle health.

This study possesses several notable strengths. It employs a robust statistical framework that integrates multiple models to comprehensively assess both the independent and joint effects of mPAEs on grip strength. mPAEs mixture exposure as a key contributor to the negative association between grip strength represents a novel finding that extends existing literature. Additionally, focusing on the Chinese Guangzhou population provides valuable insights into this specific demographic context, underscoring the need for further large-scale prospective research in diverse populations to validate and expand upon these findings. Meanwhile, a limitation of this study is its cross-sectional design, which restricts our ability to infer causality between mPAEs exposure and reduced grip strength. The simultaneous measurement of exposure and outcome raises the possibility of reverse causality, where lower grip strength, potentially due to underlying health conditions or reduced physical activity, could lead to behaviors or conditions (e.g., increased use of phthalate-containing products) that elevate mPAEs exposure (13). Additionally, unmeasured or inadequately controlled confounding factors, such as lifestyle variables (e.g., diet, occupational exposures, or exercise habits), may influence both mPAEs exposure and grip strength, potentially biasing our observed associations. Occupational settings have been associated with elevated urinary concentrations of phthalate metabolites, including mBzP and MBP (37). The absence of occupational exposure data in our study may have introduced residual confounding, as participants with high-exposure occupations could have different mPAEs profiles that influence grip strength outcomes. Considering the short half-lives of phthalates, using a single spot urine measurement to assess mPAEs exposure might not reliably reflect the long-term exposure situation. Such misclassification is likely to bias effect estimates toward the null, underestimating the true association between mPAEs and grip strength, as misclassified exposures dilute the observed relationships. For instance, individuals with healthier lifestyles might have lower exposure to phthalates through reduced use of personal care products and higher grip strength due to regular physical activity, confounding the exposure-outcome relationship (8, 24). This study is the potential for Type I errors due to multiple testing with nine mPAEs, which increases the risk of false-positive findings. However, given the exploratory nature of our study, which aims to identify potential associations between mPAEs mixtures and grip strength for further investigation, we chose not to apply Bonferroni correction in the primary analyses to avoid overly conservative results that might obscure meaningful patterns, especially in the context of mixture models like BKMR and WQS. To address these limitations, future research should employ prospective designs to track changes in mPAEs exposure before and after changes in grip strength. Additionally, incorporating detailed lifestyle data (e.g., through validated questionnaires on product use, diet, and physical activity), occupational exposure assessments and biomarkers of confounding factors (e.g., nutritional status) would help control for unmeasured confounding.

## 5 Conclusion

In summary, this cross-sectional study identified significant associations between various mPAEs—including monomethyl phthalate (mMP), mono-isobutyl phthalate (miBP), and mono (2-ethylhexyl) phthalate (mEHP)—and decreased grip strength in the Chinese population. The analysis of mixed mPAEs exposure revealed a negative correlation between co-exposure to mPAEs and grip strength. These findings enhance our understanding of the impact of co-exposure to mPAEs on grip strength and underscore the importance of considering multiple phthalate metabolites simultaneously in future research.

## Data Availability

The raw data supporting the conclusions of this article will be made available by the authors, without undue reservation.
