# Supplementary material for: Association between combined urinary phthalate metabolites exposure and grip strength among residents in Guangzhou, China
Source: Front Public Health. 2025 May 30;13:1545872. doi: 10.3389/fpubh.2025.1545872 (PMC12162582; doi:10.3389/fpubh.2025.1545872)
Supplement: Supplementary file 1 [file Table_1.docx]

Supplementary Table S1. The association between mPAEs and grip strength

| mPAEs  (μg/g creatinine) | Mean | SD | Percentile | | | | |
| --- | --- | --- | --- | --- | --- | --- | --- |
|  |  |  | P5 | P25 | P50 | P75 | P95 |
| mMP | 11.03 | 13.55 | 1.93 | 3.98 | 7.00 | 13.27 | 33.36 |
| mEP | 20.80 | 35.16 | 1.76 | 4.18 | 8.36 | 20.40 | 83.61 |
| miBP | 82.41 | 95.15 | 18.41 | 31.94 | 53.64 | 96.19 | 232.58 |
| mnBP | 150.78 | 204.12 | 13.92 | 47.34 | 87.62 | 164.91 | 485.55 |
| mCHP | 0.43 | 0.84 | 0.03 | 0.05 | 0.09 | 0.37 | 2.10 |
| mEOHP | 22.92 | 31.94 | 3.19 | 7.03 | 13.08 | 26.09 | 69.80 |
| mEHHP | 29.37 | 38.78 | 4.92 | 9.28 | 17.50 | 34.25 | 86.37 |
| mBzP | 0.63 | 1.66 | 0.03 | 0.08 | 0.16 | 0.44 | 3.01 |
| mEHP | 13.17 | 17.10 | 1.02 | 4.24 | 7.91 | 15.90 | 41.05 |
